# An Analytical Model for BDS B1 Spreading Code Self-Interference Evaluation Considering NH Code Effects

**DOI:** 10.3390/s17040663

**Published:** 2017-03-23

**Authors:** Xin Zhang, Xingqun Zhan, Shaojun Feng, Washington Ochieng

**Affiliations:** 1 School of Aeronautics and Astronautics, Shanghai Jiao Tong University, Shanghai 200240, China; xqzhan@sjtu.edu.cn; 2 Centre for Transport Studies, Imperial College London, London SW7 2BU, UK; s.feng@ic.ac.uk (S.F.); w.ochieng@ic.ac.uk (W.O.)

**Keywords:** BDS, short spreading code, high-sensitivity, spectral separation coefficient, self-interference, Neumann-Hoffmann

## Abstract

The short spreading code used by the BeiDou Navigation Satellite System (BDS) B1-I or GPS Coarse/Acquistiion (C/A) can cause aggregately undesirable cross-correlation between signals within each single constellation. This GPS-to-GPS or BDS-to-BDS correlation is referred to as self-interference. A GPS C/A code self-interference model is extended to propose a self-interference model for BDS B1, taking into account the unique feature of the B1-I signal transmitted by BDS medium Earth orbit (MEO) and inclined geosynchronous orbit (IGSO) satellites—an extra Neumann-Hoffmann (NH) code. Currently there is no analytical model for BDS self-interference and a simple three parameter analytical model is proposed. The model is developed by calculating the spectral separation coefficient (SSC), converting SSC to equivalent white noise power level, and then using this to calculate effective carrier-to-noise density ratio. Cyclostationarity embedded in the signal offers the proposed model additional accuracy in predicting B1-I self-interference. Hardware simulator data are used to validate the model. Software simulator data are used to show the impact of self-interference on a typical BDS receiver including the finding that self-interference effect is most significant when the differential Doppler between desired and undesired signal is zero. Simulation results show the aggregate noise caused by just two undesirable spreading codes on a single desirable signal could lift the receiver noise floor by 3.83 dB under extreme *C*/*N*_0_ (carrier to noise density ratio) conditions (around 20 dB-Hz). This aggregate noise has the potential to increase code tracking standard deviation by 11.65 m under low *C*/*N*_0_ (15–19 dB-Hz) conditions and should therefore, be avoided for high-sensitivity applications. Although the findings refer to Beidou system, the principle weakness of the short codes illuminated here are valid for other satellite navigation systems.

## 1. Introduction

The BeiDou Navigation Satellite System (BDS) is a relatively new member of the Global Navigation Satellite System (GNSS) family. Its global role has recently been recognized by formal acceptance by the International Maritime Organization (IMO) as a key part of its World-Wide Radio-Navigation System (WWRNS). This is in addition to the adoption of 1575.42 MHz for the future B1c component (instead of 1561.098 MHz currently used by B1 signal) for interoperability with other GNSS’s. For both mass-market and professional GNSS users, BDS will provide improved accuracy, integrity, continuity and availability. There are several factors that determine a GNSS receiver’s tracking sensitivity, one of which is self-interference. This is due to cross-correlation between codes from other satellites, within the same GNSS constellation. Self-interference problem is present in GNSS with short (1023 or 2046 chips in one code period) spreading codes. The fact that this problem has little influence on existing GNSS such as GPS indicates it is not a major problem, but in some exceptional cases when signal is extremely weak (down to 20 dB-Hz or even weaker), this could cause trouble for receiver correlators, which requires close investigation. Take acquisition for example, the effects could be two-fold. First, the self-interference between a strong and a weak signal may surpass the weak signal autocorrelation peak. Second, the very same self-interference contributes to the pre-detection noise floor, effectively reducing the processing gain of a weak signal; this could amount to a 9 dB processing gain degradation in weak signal acquisition [1]. In addition, in reseach such as multiple access capacity of GNSS, self-interference is a non-negligible factor when considering the upper limit of navigation satellites in the sky [2]. All these examples necessitates evolution of existing self-interference evaluation methods.

Since GPS achieved full operational capability (FOC), research and development activities on the understanding and mitigation of self-interference were historically focused on GPS L1 C/A code. Typically, there are two methods to study this effect: simulation or an analytical way. Simulation can provide highly accurate results but at a cost in terms of computation time. A single location on earth surface takes about 11 CPU hours to gather the data for a whole day: 30-s data sets spaced every 15 min [3], and this translates into 13.752 CPU seconds for each second of simulated data. The accuracy of the simulation approach is 0.5 degrees (1 σ) for carrier tracking standard deviation, with coherent integration time equal to 0.02 s [4]. The analytical method is expected to achieve a lower but acceptable accuracy with a much reduced computation overhead. This paper intends to introduce a method for BDS self-interference evaluation on top of existing GPS self-interference models.

The analytical model of GPS L1 C/A self-interference has long been investigated. Self-interference when introduced as extra noise injected into a receiver, like cross correlation between different GNSS’s, is evaluated using spectrum separation coefficient (SSC) [5,6]. It is common practice that analytical models treat the short code as a simple wide sense stationary (WSS) random process using a single parameter auto-correlation function (ACF) [7]. However, this simple model for C/A code self-interference ignores the fact that the spreading code repeats every 1023 chips and treats C/A code as an infinite long sequence of random plus/minus ‘1’ s. Efforts to improve the accuracy of the analytical model were made by Dierendonck and Hegarty [8], Hegarty and Dierendonck [9], Shibata and Maeda [10] and Dierendonck et al. [11]. More complex WSS models were derived to predict C/A code self-interference by considering the real L1 C/A signal comprised of 20 repetitions (which was previously ignored) of spreading code of 1ms long in one navigation data bit. In fact, the short spreading codes including the GPS L1 C/A code (and also BDS B1-I code), are more accurately modelled as a cyclostationary random process using two-parameter ACF [12]. Although simple, the existing analytical methods generate results worse than (typically with accuracy of 0.2 degrees (1 σ) for carrier tracking standard deviation over a 10-s sliding window) or even different from those from pure simulation methods using a constellation simulator, a software receiver, and a software signal generator [13]. In addition, to date all analytical models on self-interference are for GPS L1 C/A code, and there are no models for self-interference within the BDS constellation.

To evaluate BDS self-interference, a simple analytical model is developed from an existing model for GPS self-interference [12] to predict the impact of B1-I code self-interference on BDS receiver functions that are dependent primarily on the sum of the correlations (e.g., carrier phase tracking and data demodulation). The focus is on self-interference between BDS MEO and IGSO satellites. Self-interference effects between BDS GEOs are not studied because GEO signals do not interfere with MEO signals due to the high symbol rate [14]. The effects of the NH code [15], only present in the BDS MEO and IGSO B1-I signal, on self-interference evaluation, is addressed during the development of the analytical model.

The proposed model is based on the perspective that self-interference can be modelled by the introduction of an extra white noise in receiver operations. The noise has the same effects on code and carrier tracking as self-interference. The accuracy of the model is ensured by using a two-parameter ACF to characterize a B1-I code either with or without navigation data. This type of cyclostationary analytical model will be validated as an accurate method to evaluate self-interference for short codes, producing excellent agreement with actual receiver observables, using hardware simulator intermediate frequency (IF) data and a software receiver.

The Section “Signal and Correlator Model” provides basic assumptions and signal models to be used in the development of the proposed analytical model. Section “SSC and Equivalent White Noise Level” derives analytical models for the variance at the correlator output for three situations: the data bits of the desired and undesired signals are (1) aligned or (2) misaligned by an integer number of code chips or (3) misaligned by a fraction of a code chip. These three models are the basis of the proposed analytical model. Section “Proposed Model for BDS MEO/IGSO B1-I Self-Interference” presents the proposed model based on SSCs derived in the previous section. Section “Model Validation” demonstrates the validity of the model using live BDS data record and a modified software receiver. Section “Results” analyzes typical behavior of B1-I self-interference and its impact on receiver range observables using the developed model. Section “Conclusions” summarizes the expected contributions and presents a framework for further efforts.

## 2. Signal and Correlator Model

**Figure 1:**
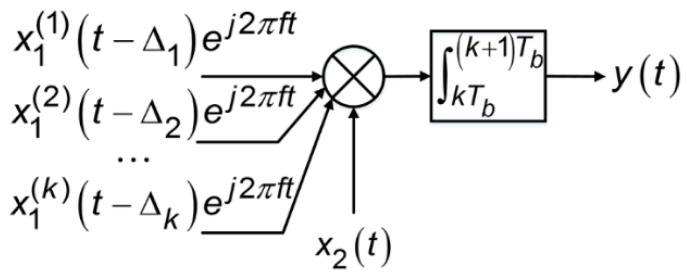
Generic correlator model.

Figure 1 shows a generic correlator model, whose input is the undesired (interfering) signal, *x_1_^(k)^*(*t* − Δ*_k_*)*e^j^*^2*πf*^, and the desired (victim) signal is *x*_2_(*t*). There is a differential Doppler frequency, i.e., difference between Doppler frequencies, denoted as *f*, between the undesired and desired signals. These two signals are correlated by mixing and integration over an arbitrary navigation data bit period, [*kT_b_*, (*k* + 1)*T_b_*), where *T_b_* = 20 ms and *k* = 0, 1, 2, ....

To simplify the problem without loss of generality, we focus on an individual undesired signal including a specific spreading code identified by PRN (Pseudo Random Noise) number, *x*_1_(*t*). This undesired signal can be any one of the undesired signals *x*_1_*^(k)^*(*t*) in the input to the correlator in Figure 1. Therefore, the undesired signal without Doppler or time delay is modelled as:

(1) $$x_{1}\left(t\right)=\sum_{k=-\infty }^{\infty }d_{k}\sum_{u=0}^{19}\alpha_{u}\sum_{v=0}^{2045}c_{v}p_{T_{c}}\left(t-\left[40,920k+2046u+v\right]T_{c}\right)$$

where *p_Tc_*(t) is a rectangular pulse of width *T_c_*:

(2) $$p_{T_{c}}\left(t\right)=\{\begin{array}{cc} 1 & 0\leq t<T_{c}\\ 0 & else \end{array}$$

and *c_v_* ∈ {−1, 1} is spreading code chips with a chipping rate 1/*T_c_* = 2.046 MHz. The chip width is *T_c_*; *d_k_* is navigation data bits with bit rate 1/*T_b_* = 50 bps, and the bit width is *T_b_*. *α_u_* is the NH code adopted for B1-I signal and takes a fixed pattern of (0, 0, 0, 0, 0, 1, 0, 0, 1, 1, 0, 1, 0, 1, 0, 0, 1, 1, 1, 0), which spans one navigation data bit. The spreading code here is treated as a random periodic sequence of length 2046 chips, with chips independent from each other. The navigation data bit stream is also treated as a random periodic sequence, but with an infinite length.

**Figure 2:**
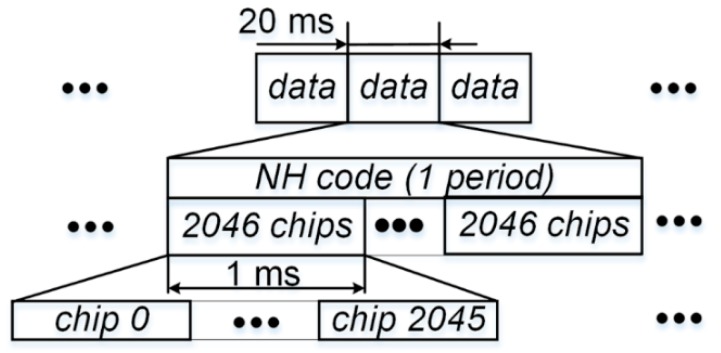
Spreading code, navigation data bit, and NH code relationship for B1-I.

In order to facilitate the development in the appendices, spreading code period, *T*, is introduced, and there is an obvious relationship among *T_c_*, *T*, and *T_b_*:*T_b_* = 20*T* = 40,920*T_c_*. This relationship is illustrated in Figure 2. The receiver local code replica or desired signal is modelled similarly as:

(3) $$x_{2}\left(t\right)=\sum_{k=-\infty }^{\infty }\sum_{l=0}^{2045}c_{k}p_{T_{c}}\left(t-\left[2046k+l\right]T_{c}\right)$$

using the same notation as Equation (1). The only difference between the desired and undesired code is that the undesired one has a time delay of ∆ seconds, which is normally within the range of ±20 ms for BDS users on or near the horizon of the Earth. Both signals are cyclostationary, not WSS, and therefore, their characteristics are accurately represented using a two-parameter ACF [16]. Since the period of cyclostationarity is 20 ms, the model derived in the following sections only takes into account time delays ranging from 0 to 20 ms, i.e., 0 ≤ ∆ ≤ 20 ms, which is equal to the time delay modulo 20 ms.

In a real receiver, both the incoming signal and local replica have a Doppler shift. However, for simplicity and without loss of generality, we assume here that only the incoming signal has its Doppler shift, *f*, while the local replica has zero Doppler. This is simply because the receiver carrier tracking loop is after all, tracking the *differential* Doppler between the incoming and the local replica, not the absolute Doppler of each signal.

## 3. SSC and Equivalent White Noise Level

The extent of the undesired signal’s impact on the desired signal is characterized by an equivalent white noise level, which is derived from SSC, whose analytical forms are derived in this section. The *k*-th correlator output, *y_k_*, is a random variable. Its mean value is:

(4) $$E\left\{y_{k}\right\}=\int_{kT_{b}}^{\left(k+1\right)T_{b}}E\left\{x_{1}\left(t-\Delta \right)\right\}E\left\{x_{2}\left(t\right)\right\}e^{j2\pi ft}dt=0$$

and its variance is:

(5) $$\begin{array}{l} E\left\{{\left|y_{k}\right|}^{2}\right\}=E\left\{y_{k}\cdot y_{k}^{*}\right\}\\ =E\left\{\int_{kT_{b}}^{(k+1)T_{b}}\int_{kT_{b}}^{(k+1)T_{b}}x_{1}\left(s-\Delta \right)x_{1}\left(t-\Delta \right)x_{2}\left(s\right)x_{2}\left(t\right)e^{j2\pi fs}e^{-j2\pi ft}dsdt\right\}\\ =\int_{kT_{b}}^{(k+1)T_{b}}\int_{kT_{b}}^{(k+1)T_{b}}E\left\{x_{1}\left(s-\Delta \right)x_{1}\left(t-\Delta \right)\right\}E\left\{x_{2}\left(s\right)x_{2}\left(t\right)\right\}e^{j2\pi fs}e^{-j2\pi ft}dsdt\\ =\int_{kT_{b}}^{(k+1)T_{b}}\int_{kT_{b}}^{(k+1)T_{b}}R_{11}\left(s-\Delta ,t-\Delta \right)R_{22}\left(s,t\right)e^{j2\pi fs}e^{-j2\pi ft}dsdt \end{array}$$

where the dot within the curly brace means “dot product”. The development of Equation (5) uses the definition of auto-correlation function of two complex signals. The two-parameter ACF’s of both the undesired signal and receiver local replica are derived in Appendix A. The result is as follows:

(6) $$R_{22}\left(s,t\right)=\sum_{p=-\infty }^{\infty }p_{T_{c}}\left(t-2046pT_{c}-vT_{c}\right)$$

where *R*_22_(*s*, *t*) is the two-parameter ACF of the local replica, the integer *v* is selected as:

(7) $$v=\mathrm{mod}\left(\mathrm{floor}\left(s/T_{c}\right),2046\right)$$

and:

(8) $$R_{11}\left(s-\Delta ,t-\Delta \right)=\sum_{p=0}^{19}p_{T_{c}}\left(t-\Delta -\left[40,920k+2046p+v\right]T_{c}\right)$$

where *R*_11_(*s*, *t*) is the two-parameter ACF of the undesired signal, ∆ is the time delay of the undesired signal and the integer *k* and *v* are selected as:

(9) $$k=\mathrm{floor}\left(\left(s-\Delta \right)/T_{b}\right)$$

(10) $$v=\mathrm{mod}\left(\mathrm{floor}\left(\left(s-\Delta \right)/T_{c}\right),2046\right)$$

where function mod(*a*, *m*) returns the remainder after division of *a* by *m* where *a* is the dividend and *m* is the divisor. Function floor(*x*) rounds *x* to the nearest integer less than or equal to *x*.

The development of Equations (8)–(10) has taken into account the special structure of the B1-I signal which has an NH code modulated on it: in subsection “Desired signal ACF” of Appendix A it can be seen that since the NH code is a fixed pattern finite-length code, it is treated as a deterministic variable here.

**Figure 3:**
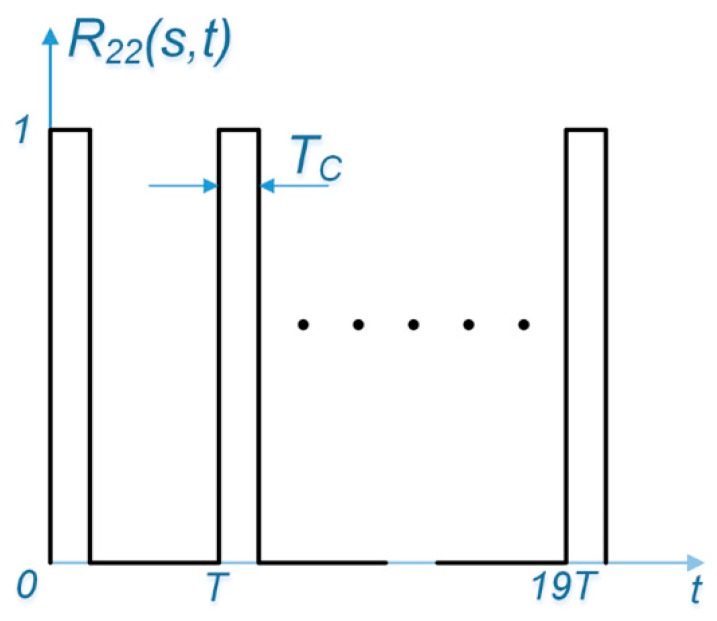
ACF for the undesired signal, which is a pulse train of 20 pulses, each of which has a width of *T_c_*. Note that this figure is not to scale.

Figure 3 illustrates an example of ACF for the desired signal *R*_22_(*s*, *t*) with *s* ∈ [0, *T_c_*). For every fixed value of the first parameter, *s*, this two parameter ACF is composed of 20 pulses of width *T_c_* with unity amplitude, over arbitrary 20-ms correlation interval, which is equal to one data bit width. For example, if *s* falls within the time duration of the repetition of the first chip of the spreading code sequence, the ACF takes on a value of unity during each repetition of this chip and zero elsewhere. The same ACF results if *s* fell within repetition of the first chip, e.g., *s* ∈ [7*T*, 7*T*+*T_c_*). The ACF for the undesired signal can be obtained by shifting the desired signal’s ACF by ∆.

The rest of this section is divided into three subsections. Analytical model for variance at the correlator output under three circumstances are derived: (i) data bits of the undesired and desired signal are aligned, (ii) data bits of these two signals are not aligned by an integer multiples of a code chip, and (iii) data bits of these two signals are misaligned by an arbitrary delay. This variance is then converted to the equivalent white noise level using SSC, which is the metric to measure how the self-interference affects receiver performance [17].

### 3.1. Data Bits Aligned

**Figure 4:**
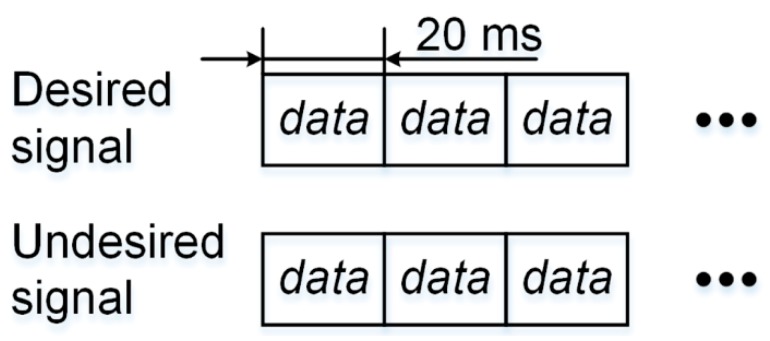
Undesired and desired signals with navigation data bits perfectly aligned.

When the data bits modulated on the undesired and desired signal are aligned, i.e., ∆ = 0, the situation is illustrated in Figure 4.

In this case, ACF’s of the undesired and desired signals become identical over any arbitrary integration interval. The variance of the correlator output could be represented as:

(11) $$\begin{array}{ll} E\left\{{\left|y_{k}\right|}^{2}\right\} & =\int_{kT_{b}}^{(k+1)T_{b}}\int_{kT_{b}}^{(k+1)T_{b}}R_{11}\left(s,t\right)R_{22}\left(s,t\right)e^{j2\pi fs}e^{-j2\pi ft}dsdt\\ & =\int_{kT_{b}}^{(k+1)T_{b}}\int_{kT_{b}}^{(k+1)T_{b}}R_{22}\left(s,t\right)e^{j2\pi fs}e^{-j2\pi ft}dsdt \end{array}$$

the last line of which comes from the fact that the ACF’s of the incoming and local code are identical: both of them are unity pulse trains. As derived in Appendix B, substituting Equation (6) into Equation (11), we have:

(12) $$\begin{array}{ll} E\left\{{\left|y_{k}\right|}^{2}\right\} & =TT_{c}{\left[\frac{\sin\left(\pi fT_{c}\right)}{\left(\pi fT_{c}\right)}\frac{\sin\left(\pi fT_{b}\right)}{\left(\pi fT\right)}\right]}^{2}\\ & ={\left(T_{b}\right)}^{2}\frac{T_{c}}{T}{\left[\sin c\left(fT_{c}\right)\sin c\left(fT_{b}\right)\right]}^{2} \end{array}$$

where:

(13) $$\sin c\left(t\right)≜\{\begin{array}{ll} 1 & t=0\\ \frac{\sin\left(\pi t\right)}{\pi t} & t\neq 0 \end{array}$$

is the sampling function. On the other hand, assume that the correlator shown in Figure 1 was driven by additive Gaussian white noise with power spectral density *I*_0_, then its output should have zero mean and a variance of:

(14) $$E\left\{{\left|y_{k}\right|}^{2}\right\}≜I_{0}T_{b}$$

which is the product of the power spectral density and equivalent bandwidth 1/*T_b_*. Comparing Equations (12) and (14), and an equivalent white noise with power spectral density *I*_0_ is obtained defined as:

(15) $$I_{0}=\frac{E\left\{{\left|y_{k}\right|}^{2}\right\}}{T_{b}}=\frac{T_{c}}{T}T_{b}{\left[\sin c\left(fT_{c}\right)\sin c\left(fT_{b}\right)\right]}^{2}$$

because this equivalent white noise generates the same variance values as a true interference does at the output of the correlator. Based on this definition, we can further define the spectral separation coefficient [17] for B1-I as *SSC_B_*_1-*I*_:

(16) $$\begin{array}{ll} I_{0} & ≜P_{R}\cdot \frac{T_{c}}{T}T_{b}{\left[\sin c\left(fT_{c}\right)\sin c\left(fT_{b}\right)\right]}^{2}\\ & ≜P_{R}\cdot SSC_{B1-I} \end{array}$$

where, *P_R_* is inserted for the more general case where an undesired signal has an arbitrary power level in watts.

### 3.2. Data Bits Misaligned by an Integer Number of Code Chips

**Figure 5:**
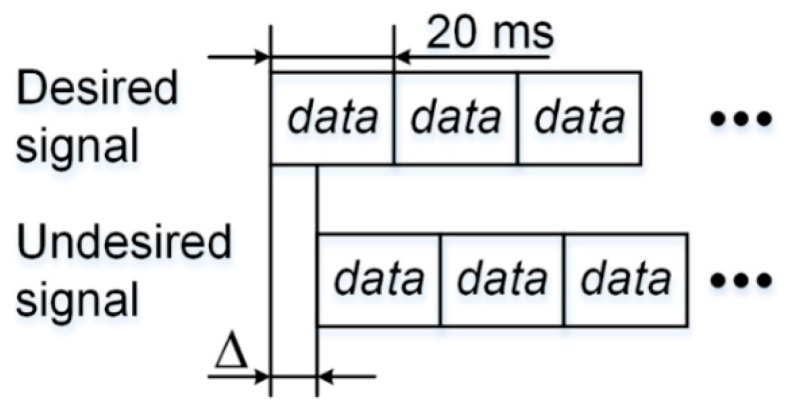
Undesired and desired signals misaligned by ∆.

When the data bits modulated on the undesired and desired signals are aligned, i.e., ∆ is not zero, the situation is illustrated in Figure 5.

In this case, the ACF of the incoming/undesired signal is:

(17) $$R_{11}\left(s-\Delta ,t-\Delta \right)R_{22}\left(s,t\right)=\{\begin{array}{ll} \sum_{p=0}^{19}p_{T_{c}}\left(t-\Delta -\left(40,920k+2046p+v\right)T_{c}\right) & \;0\leq s<\Delta \\ \sum_{p=0}^{19}p_{T_{c}}\left(t-\Delta -\left(2046p+v\right)T_{c}\right) & \Delta \leq s<T_{b} \end{array}$$

where *k* and *v* is set up using Equations (7), (9) and (10). As derived in Appendix B, assuming that the data bit misalignment is an integer multiple of the spreading code period *T*, i.e., ∆ = *KT* for 0 *≤* *K* < 20, where *K* is an integer, results in:

(18) $$SSC_{B1-I}=\frac{TT_{c}}{T_{b}}{\left[\sin c\left(fT_{c}\right)\right]}^{2}\left[\frac{\sin^{2}\left(\pi fKT\right)+\sin^{2}\left(\pi f(20-K)T\right)}{\sin^{2}\left(\pi fT\right)}\right]$$

which, for users near or at the Earth’s horizon, could be approximated as:

(19) $$SSC_{B1-I}≃\frac{TT_{c}}{T_{b}}\left[\frac{\sin^{2}\left(\pi fKT\right)+\sin^{2}\left(\pi f(20-K)T\right)}{\sin^{2}\left(\pi fT\right)}\right]$$

More generally, assuming that the data bit misalignment is an integer multiple of the spreading code chip width *T_c_*, i.e., ∆ = *KT* + *CT_c_*, where 0 *≤* K < 20, 0 < C < 2046, *K* and *C* is an integer, yields:

(20) $$SSC_{B1-I}=\frac{2046-C}{2046}SSC_{B1-I,K}+\frac{C}{2046}SSC_{B1-I,K+1}$$

where *SSC_B_*_1-*I*,*K*_ is the SSC in Equation (18) for any users or SSC in Equation (19) for users near or at the Earth’s horizon, when ∆ = *KT*.

### 3.3. Data Bits Misaligned by a Fraction of a Code Chip

Section 3.2 “*Data Bits Misaligned by an Integer Number of Code Chips*” summarizes SSC expressions that is valid when the differential time delay between undesired and desired signals, ∆ is multiples of a single code chip *T_c_*. With this constraint, *R*_11_(*s* − ∆, *t* − ∆)*R*_22_(*s*,*t*) is a pulse train of width *T_c_*, given by Equation (17).

**Figure 6:**
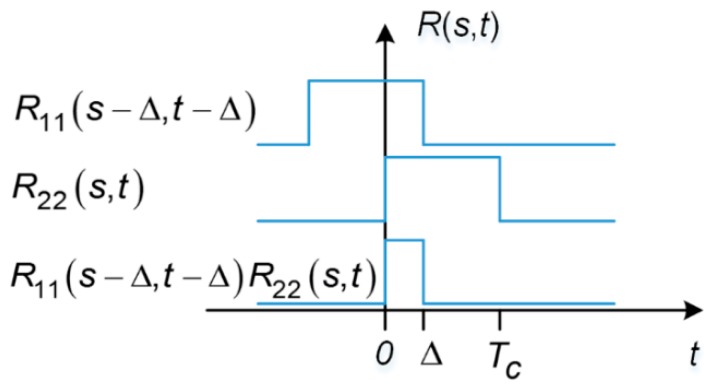
One repetition of R_11_(s − ∆, t − ∆)R_22_(s,t) as the result of data bit misalignment, with 0 ≤ s < ∆.

**Figure 7:**
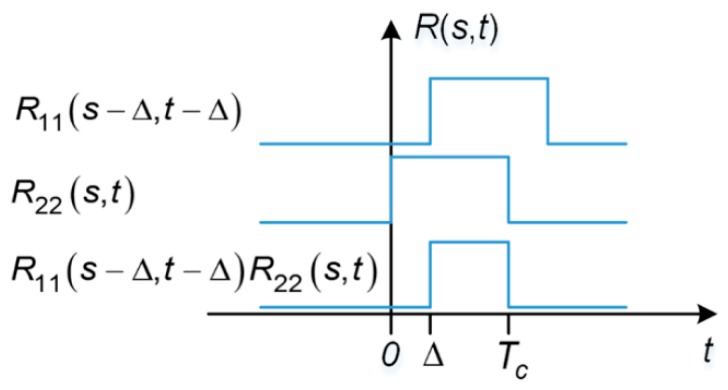
One repetition of R_11_(s − ∆, t − ∆)R_22_(s,t) as the result of data bit misalignment, with ∆ ≤ s < *T_c_*.

Now we take a step further to examine what the SSC should look like when ∆ is only a fraction of a code chip. In this case, *R*_11_(*s* − ∆, *t* − ∆)*R*_22_(*s*,*t*) is still a pulse train of uniformed width, but the pulse width is no longer equal to *T_c_*. As illustrated in Figure 6 and Figure 7, for fixed *s*, *R*_11_(*s* − ∆, *t* − ∆)*R*_22_(*s*,*t*) is either a train of pulses of width ∆ or *T_c_* − ∆.

When the differential time delay takes on arbitrary value, we proceed by using *τ* = mod(∆, *T_c_*), 0 *≤* *τ < T_c_*. Therefore, *R*_11_(*s* − ∆, *t* − ∆)*R*_22_(*s*,*t*) will be a train of pulses of width *τ* for *τ/T_c_* percent of *T_c_*, or a train of pulses of width *T_c_* – *τ* for (*T_c_* – *τ*)/*T_c_* percent of *T_c_*. The total effect of such arbitrary time delay, for the differential Doppler, is that a factor of *T_c_* in the *SSC_B_*_1-*I*_ expressions in the Section 3.2 “*Data Bits Misaligned by an Integer Number of Code Chips*” is replaced by [12]:

(21) $$\frac{\tau }{T_{c}}\cdot \tau +\frac{\left(T_{c}-\tau \right)}{T_{c}}\cdot \left(T_{c}-\tau \right)$$

which implies the expectation of receiving such a time difference. It can be seen from Equation (21) that spreading code self-interference is maximized when *τ =* 0, i.e., when the misalignment between the desired and undesired signals happens to be multiples of a code chip; it can be seen also that self-interference is minimized then *τ = T_c_/*2. Moreover, the average of Equation (21) can be computed as:

(22) $$\frac{1}{T_{c}}\int_{0}^{T_{c}}\left[\frac{\tau^{2}}{T_{c}}+\frac{{\left(T_{c}-\tau \right)}^{2}}{T_{c}}\right]d\tau =\frac{2}{3}T_{c}$$

which, when substituted for *T_c_* in Equation (19) yields:

(23) $$SSC_{B1-I}≃\frac{2}{3}\frac{TT_{c}}{T_{b}}\left[\frac{\sin^{2}\left(\pi fKT\right)+\sin^{2}\left(\pi f(20-K)T\right)}{\sin^{2}\left(\pi fT\right)}\right]$$

which is used to compute SSC in the following sections.

## 4. Proposed Model for BDS MEO/IGSO B1-I Self-Interference

Based on Equations (20) and (23), the proposed model for BDS MEO/IGSO B1-I self-interference, or the aggregate equivalent white noise due to self-interference can be expressed as:

(24) $$\begin{array}{l} I_{0,m}\\ =p_{m}+\sum_{\begin{array}{l} i=1\\ i\neq m \end{array}}^{n}{\left(SSC_{B1-I}\right)}_{m}^{i}\\ =p_{m}+\frac{2T_{c}^{2}}{3T_{b}\sin^{2}\left(\pi f_{m}^{i}T\right)}\sum_{\begin{array}{l} i=1\\ i\neq m \end{array}}^{n}\{\left(2046-C_{m}^{i}\right)\left[\sin^{2}\left(\pi f_{m}^{i}K_{m}^{i}T\right)+\sin^{2}\left(\pi f_{m}^{i}\left[20-K_{m}^{i}\right]T\right)\right]\\ +C_{m}^{i}\left[\sin^{2}\left(\pi f_{m}^{i}\left[K_{m}^{i}+1\right]T\right)+\sin^{2}\left(\pi f_{m}^{i}\left[19-K_{m}^{i}\right]T\right)\right]\} \end{array}$$

where *I*_0,*m*_ is the aggregate equivalent white noise imposed on the *m*-th signal by self-interference, *p_m_* is the received power at the antenna, (*SSC_B_*_1-*I*_)*^i^_m_* is the SSC between the *i*-th and *m*-th signals, which is determined by differential Doppler *f^i^_m_* and differential time delay *K^i^_m_ T* + *C^i^_m_ T_c_*.

Table 1 shows an example of how to use the developed analytical model, i.e., Equations (20) and (23) to predict *SSC_B_*_1-*I*_ and equivalent white noise *I*_0_.

Note that the last line of Table 1 comes from the sum of *I*_0_ of PRN 8 and 9. At a specific time, there are 8 satellites visible to the receiver. In Table 1, the first column is the PRN number. The second column is the relative received power, relative to −160 dBW. The third column is the true (geometric) range from satellite to the receiver. The fourth column is the true Doppler. These four columns are the parameters used to compute the fifth and sixth columns, i.e., *SSC_B1-I_* and equivalent white noise *I*_0_, respectively. Since here it is assumed that PRN 6 is the desired signal, it is unnecessary to compute its own *SSC* and *I*_0_.

Table 2 presents the procedure to compute *SSC* and *I*_0_. The procedure described in Table 2 is simply a re-iteration of the development of the model Equation (24).

## 5. Model Validation

Live BDS signal records from a hardware signal simulator are collected to test if the proposed model is correct within a certain boundary, set by established benchmark work.

The validity of the model is not directly proved because the proposed model includes the NH code effect and there is no current work covering this unique signal component on B1 self-interference evaluation, though there does exist effective ways to overcome NH code impact on various stages of signal processing [15]. Since no other formula is currently available, an indirect method is applied to justify the assumptions and the proposed model. Self-interference effect on receiver noise is intangible or indirect to see or to feel but can appropriately be shown and visualized by its impact in the measurement domain (i.e., DLL output or a step further, code range), and in this regard, we choose to apply Betz’s well-established expressions linking *C*/*N*_0_ with discriminator output statistics. Since no validation of Betz’s model was provided in Betz’s paper [18] or his later publications, and therefore in order to use Betz’s formula, we had first to prove it (or at least, show its effectiveness using real measurements), which is presented in this section and does form an integral part of the completeness and soundness of the validation logic.

### 5.1. Criterion

Model validity is checked by comparing the predicted and measured tracking standard deviation, using the expression [18]:

(25) $$\mathrm{var}\left\{\tau_{k}^{u}|\tau_{k}^{s}\right\}=\frac{\mathrm{var}\left\{e\left(\varepsilon \right)|\tau_{k}^{s}\right\}}{C_{s}^{4}K^{2}}$$

where *K* is code tracking loop gain and *C_s_* the measured signal power. var{*e*(*ε*) | *τ^s^_k_*} is measured discriminator output variance. var{*τ^u^_k_* | *τ^s^_k_*} is variance of the unsmoothed code delay estimate *τ^u^_k_*, which is predicted by using Equation (26) [19]:

(26) $$\mathrm{var}\left\{\tau_{k}^{u}|\tau_{k}^{s}\right\}=\frac{B_{L}\int_{-B_{r}/2}^{B_{r}/2}G_{s}\left(f\right)G_{w}\left(f\right)\sin^{2}\left(\pi f\Delta \right)df}{{\left(2\pi \right)}^{2}C_{s}{\left(\int_{-B_{r}/2}^{B_{r}/2}fG_{s}\left(f\right)\sin\left(\pi f\Delta \right)df\right)}^{2}}\left[1+\frac{1}{T{\left(\frac{C_{s}}{N_{0}}\right)}_{eff}\int_{-B_{r}/2}^{B_{r}/2}G_{s}\left(f\right)df}\right]$$

which is the analytical form of code tracking variance. The term ‘(*C_s_*/*N*_0_)*_eff_*’ is effective carrier to noise ratio, and is equal to *C_s_* − *I*_0_, where *C_s_* is the measured signal power and *I*_0_ the equivalent white noise as defined in Equation (16) and transformed into dB. Here *I*_0_ includes self-interference effects, to be evaluated using the proposed model (Equations (16) and (20)). Both *C_s_* and *I*_0_ are in dB and (*C_s_*/*N*_0_)*_eff_* should be converted back to non-logarithmic unit when it is used in Equation (26). If the model is correct, the squared root of the two sides of Equation (25) should agree within a small error tolerance for a specific channel in a software receiver:

(27) $$\left|\sqrt{\mathrm{var}\left\{\tau_{k}^{u}|\tau_{k}^{s}\right\}}-\sqrt{\frac{\mathrm{var}\left\{e\left(\varepsilon \right)|\tau_{k}^{s}\right\}}{C_{s}^{4}K^{2}}}\right|<\lambda$$

where *λ* is a tolerance value and we set it as follows. We compare our prediction accuracy with accuracy of most recent analytical methods for self-interference, such as the work by Cerruti et al. [4], where the accuracy of prediction, i.e., the difference between predicted and measured carrier tracking standard deviation is 0.5 degrees. This translates into a prediction accuracy of 0.0264 cm (0.5/360 × 19) in range by phase measurements, which is in turn, translated into code tracking prediction accuracy of 6.6 cm by a scale factor of 250, since code tracking loop is 100 (with multipath) to 250 (without multipath; this is the case with current validation settings described in Table 3, and is also the case with the benchmark work by Cerruti et al. [3]) times noisier than carrier tracking loop in terms of tracking standard deviation [20]. Therefore, we set *λ* = 0.066 m.

### 5.2. Test Setup

**Figure 8:**
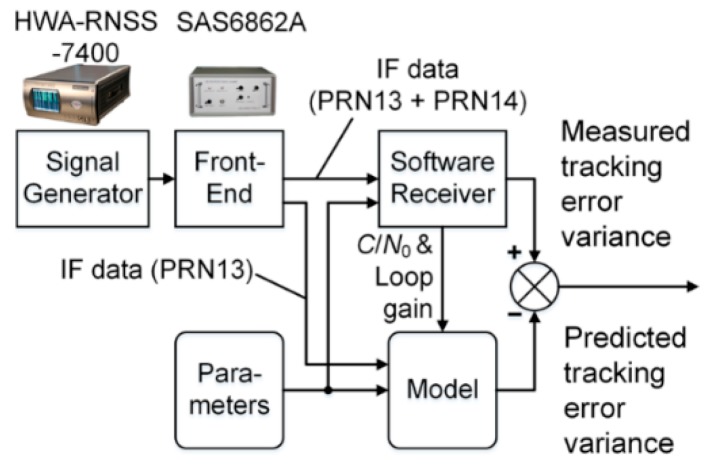
Test setup for model validation.

The test setup is illustrated in Figure 8. Since we want to compare predicted and measured tracking standard deviations, we have to collect live signal records to measure the code tracking standard deviation using a software receiver. Without loss of generality, we choose two IGSO satellites, PRN 13 and 14 instead of the whole current constellation to test the developed model. This is because: (i) In validation, only two satellites (desired and undesired signal) are necessary; (ii) PRN 13 & 14 are the two of the four currently operational IGSO satellites in BDS, and since MEO and IGSO in BDS use the same signal modulation scheme; IGSO signal will suffice to represent a combination of MEO and IGSO signals. Therefore, first we collect IF data containing both PRN 13 and PRN 14 signals and then we collect IF data containing only the PRN 13 signal. Finally, we obtain the measured code tracking standard deviation of PRN 13 using the first dataset, which includes the interference effect imposed by PRN 14. This interference effect is predicted using the estimated power (*C_s_*) from the second dataset (free of interfering signal PRN 14) and our proposed model. As a result, a hardware signal simulator and not live signals from satellites, must be used as the signal source. 

We use a HWA-RNSS-7400, which is a multi-constellation GNSS signal simulator by HWA Create Co., Ltd. (Beijing, China) [21]. It features BDS B1, B2, and B3 signal simulation and is widely used within BDS industries. We also use a SAS6862A, which is a wide-band, dual channel (GPS L1+BDS B1 and BDS B3) radio frequency front end (RFFE) with a highly stable (better than 0.005 ppm) oven controlled crystal oscillator (OCXO) [22]. We use a multi-constellation software receiver called UNIversal COmmunications and Radio Navigation receiver (UNICORN), developed by the authors at the Centre for Transport Studies (CTS), Imperial College London and used to obtain the ESA’s certificate for the “First 50 Successful Galileo Position Fixes”. The receiver has been adapted to process the B1 signal.

Parameters used to configure UNICORN and drive the analytical model are the same and are provided in Table 3. The simulation starts at UTC 00:10:00, 27 Feb 27, 2014 and two 37-s IF data was recorded using SAS6862A. The receiver is assumed to be located at 31° N, 121.5° E, at an altitude of 5 m in Shanghai, China.

UNICORN has to produce two measurements for the analytical model: *C*/*N*_0_ and code loop gain, which is indispensable for successful evaluation of tracking error variance through the model. Within UNICORN, several lines of code were inserted to calculate tracking error standard deviation.

### 5.3. Results

**Figure 9:**
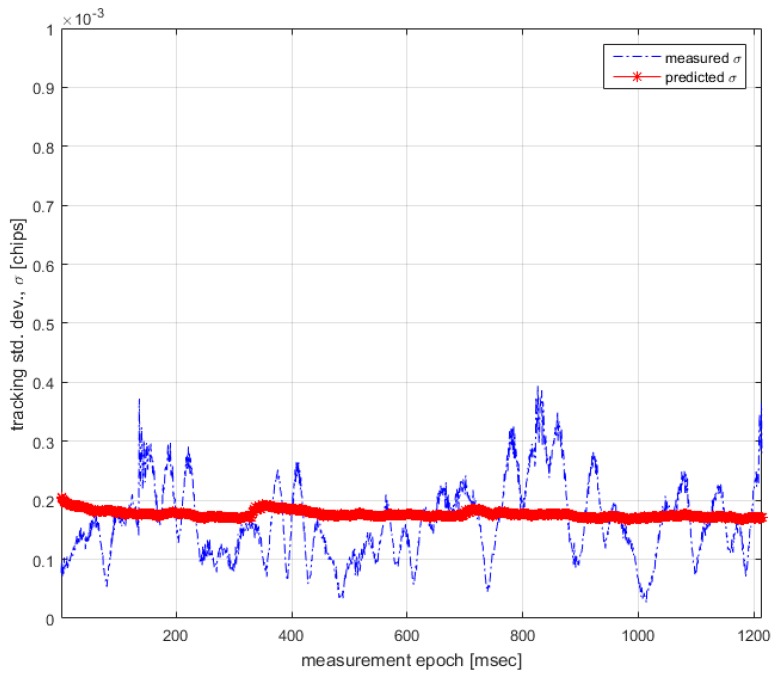
Measured vs. predicted tracking standard deviation.

Finally, the results from UNICORN and the analytical model are compared to see if the predicted values agree with measured values according to Equation (27). Figure 9 shows the results.

In Figure 9, it can be observed that the discrepancy between measured and predicted standard deviations are bounded by 0.0002 B1 primary code chips, which translates into a prediction accuracy of 0.0293 m, less than half of the threshold value of 0.66 m. This proves the validity of the analytical model.

## 6. Results

Based on the validity of the model, we now apply this model to show some insights into the typical behaviour of self-interference in BDS B1-I signals. Firstly, we try to find when the equivalent noise introduced by the self-interference approaches its maximum value.

**Figure 10:**
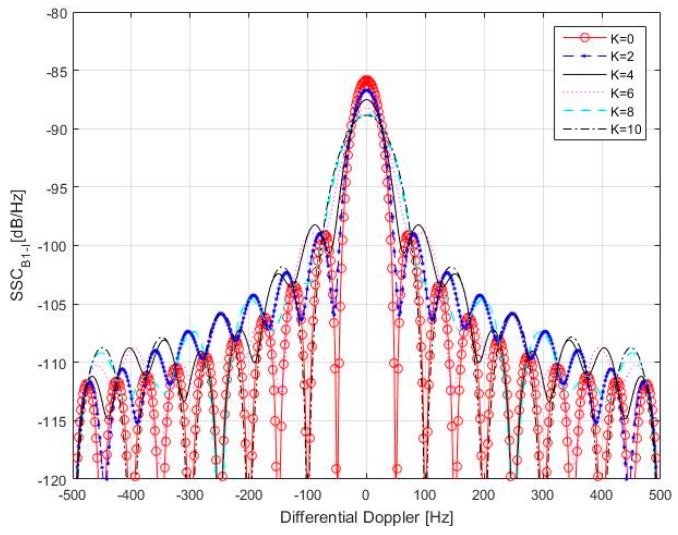
*SSC*_B1-I_ variation as a function of differential Doppler (between desired and undesired signals) and differential time delay. *K* is even.

**Figure 11:**
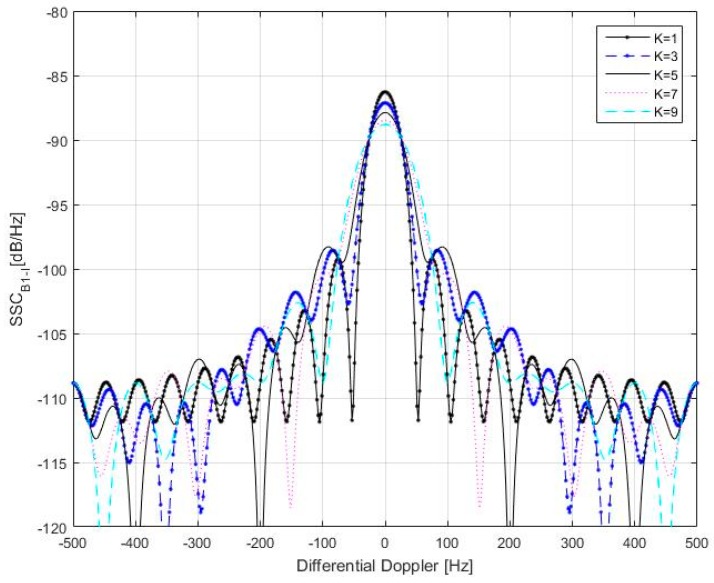
*SSC*_B1-I_ variation as a function of differential Doppler (between desired and undesired signals) and differential time delay. *K* is odd.

The SSC predicted from Equation (23) is illustrated in Figure 10 and Figure 11. Here the differential time delay between the desired and undesired signals is an integer multiple of spreading code periods. Since the SSC values for *K* > 10 is equal to SSC values with 20 − *K*, only cases with *K* *≤* 10 are shown here. To facilitate viewing, the results are shown separately when *K* is odd or even. From Figure 10 and Figure 11 we can conclude that when the differential Doppler between desired and undesired signals is bounded by 50 Hz, self-interference will introduce the most serious noise into receiver processing chain.

**Figure 12:**
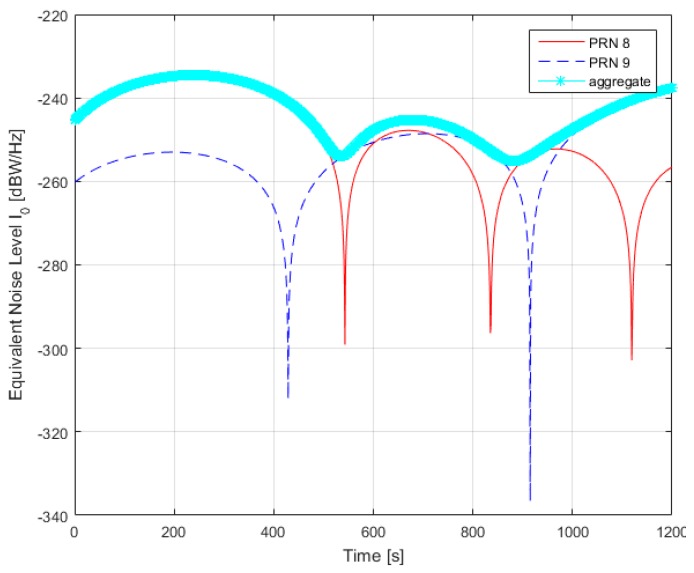
Time evolution of equivalent noise level for PRN 6, 8, and 9 in current BDS constellation.

Secondly, in order to analyze the self-interference effects in current BDS configuration, a simulation case is setup. The orbit propagation, receiver acquisition and tracking are performed by the Spirent SimGEN simulator software suite. SimGEN is capable of full and versatile case generation. Users are allowed to control multiple GNSS and regional satellite constellations including BDS, signal propagation, multipath and obstruction effects, antenna patterns, vehicle trajectory and various error models, thus vastly facilitating our simulation. For the current simulation, three inputs are needed from SimGEN, namely true range, received power, and carrier Doppler shift. However, due to the version of SimGEN, it is not able to simulate BDS constellation. The problem is bypassed by using Two Line Element (TLE) file retrieved from NORAD [23] and feeding it to the Motion control panel for GPS in SimGEN. The simulation length is 20 minutes, starting from UTCG1927, 26 April 2015, with a measurement rate of 1 Pulse Per Second (PPS). The receiver is placed in Shanghai, China. The result is shown in Figure 12. The constellation information is shown in Appendix C.

**Figure 13:**
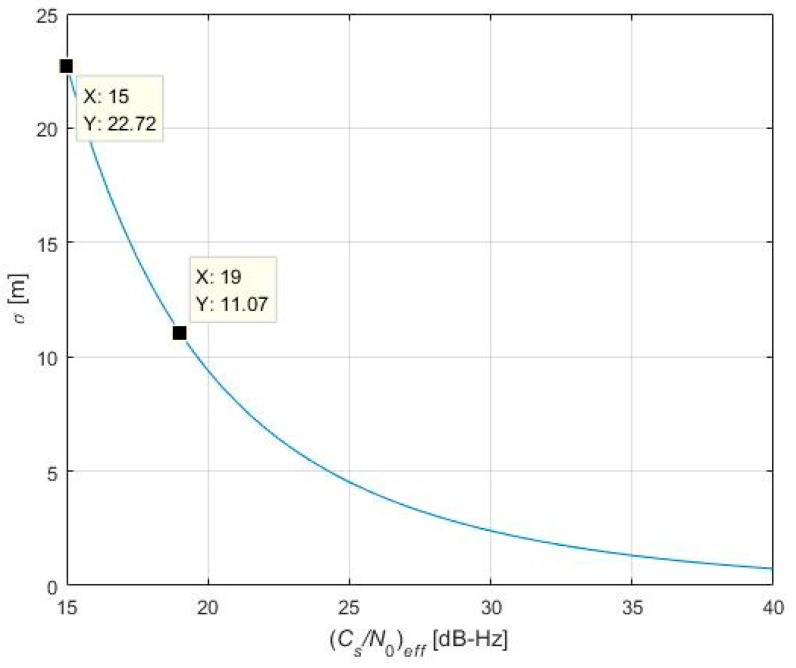
Code tracking standard deviation for different *C*/*N*_0_ values.

As illustrated in Figure 12, we can obtain such observations:

(1) Within most of the simulation interval, the individual equivalent noise and aggregate noise is below −215 dBW/Hz, which is much lower than a typical receiver noise floor of about −201.5 dBW/Hz, and therefore would not cause much trouble for most applications. Here trouble means worsening of such receiver capabilities as acquisition sensitivity and tracking jitter.
(2) During 200 and 240 s, the equivalent noise of PRN 8 and the aggregate noise reaches −200 dBW/Hz. For a typical value of receiver noise floor (−201.5 dBW/Hz), this noise floor will be transferred to −197.67 dBW/Hz. In this case, PRN 6’s effective carrier to noise density ratio (*C_s_*/*N*_0_)*_eff_* is 3.83 dB lower than the original carrier to noise density ratio, should the self-interference caused by PRN 8 be absent. This could pose a serious problem to high-sensitivity processing during acquisition, tracking or navigation bit modulation. The potential hazard caused by this (*C*/*N*_0_)*_eff_* decrease could be best exemplified by Figure 13, which shows the code tracking standard deviation versus (*C_s_*/*N*_0_)*_eff_* , using established analytical method by Zhang and Zhan [19]. The B1-I receiver used in Figure 13 is assumed to have a precorrelation bandwidth of 4 MHz, a sampling frequency of 16 MHz, and 2-bit quantization. The coherent integration interval is 20 ms and a Dot Product discriminator is used. When (*C*/*N*_0_)*_eff_* decreases from 19 to 15 dB-Hz, tracking standard deviation increases from 11.07 m to 22.72 m. In this regard, B1-I self-interference could pose a potential hazard on pseudorange measurement for high-sensitivity receivers. However, it must be underlined that for most receivers (from mass market to medium-grade receiver), a (*C*/*N*_0_)*_eff_* value under 20 dB-Hz, though may be possible, is an exceptional case. When (*C*/*N*_0_)*_eff_* decreases from 24 to 20 dB-Hz, tracking standard deviation increases from 5 m to 9 m, which does not impose significant impact on final positioning accuracy for most low- to medium-grade receivers.
(3) Around 240 s for PRN 8 and around 1200 s for PRN 9, the self-interference effect on the desired signal PRN 6 reaches its maximal value for each undesired signal respectively. This corresponds to the time when these two PRN’s Doppler shift (modulo 1 kHz) are aligned (zero differential Doppler shift) with the desired signal, PRN 6. The same situation has also been found in GPS C/A-to-C/A self-interference [4].

**Figure 14:**
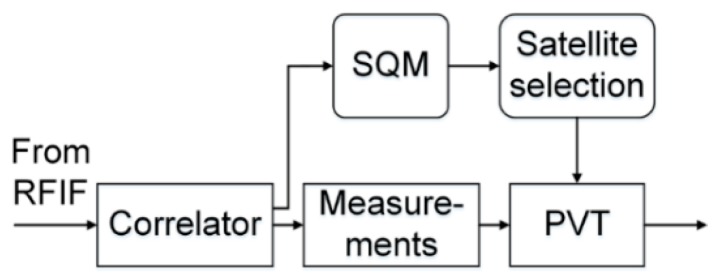
Proposed Signal Quality Monitoring (SQM) based on self-interference evaluation when selecting satellites for navigation.

In this regard, the proposed model could be used in B1 receiver as a code-tracking performance indicator, since code tracking standard deviation is directly determined by (*C_s_*/*N*_0_)*_eff_*., the effective carrier-to-noise power density ratio, which is in turn, directly affected by self-interference effects, as indicated in Figure 13. In the simplest case, the common satellite selection step (e.g., depending on DOP, Dilution of Precision) should be complemented by a new step to narrow down the list of satellites participating in navigation solution, based on the result of self-interference calculation according to Equations (20) and (23), as suggested in Figure 14.

In Figure 14, a typical receiver baseband (correlator + measurements) plus navigation module is shown, aided by a satellite selection module, whose action depends on a Signal Quality Monitoring (SQM) module [24] on top of its normal operations, monitoring the equivalent noise spectral density imposed by self-interference,. This may be an indication to receiver manufacturers for their design of strategies for satellite selection in Position, Velocity and Time (PVT) computation and signal acquisition in case of very low *C*/*N*_0_, which could be estimated stably and accurately through novel estimators [25].

## 7. Conclusions

A simple analytical model is developed to predict the impact of B1-I code self-interference on BDS receiver functions that are dependent primarily on the sum of the correlations (e.g., carrier phase tracking and data demodulation). The model is based on the fact that the code is actually a cyclostationary process rather than WSS, as previously assumed in SSC evaluation. A two-parameter ACF, in contrast to the usual one-paramter ACF, is applied to characterize B1-I code either with or without navigation data. The assumption of cyclostationarity is essential in evaluating self-interference effects for short codes, producing excellent agreement with actual receiver observables. The model is found to be able to accurately predict self-interference effects on receiver code tracking standard deviation, when the difference of time delay and Doppler between desired and undesired signals varies.

Even during the days when BDS is evolving into its Phase III stage, there is a large user group still using the legacy BDS signal. The proposed model could be used in B1 receivers as a code-tracking performance indicator and an aid to satellite selection during navigation solution.

## Figures and Tables

**Table 1:** Model utilization example.

PRN	Relative Received Power (dB)	True Range (m)	Doppler (Hz)	*SSC_B_*_1-*I*_ (dB/Hz)	*I*_0_ (dBW/Hz)
66	11.067	36,868,693	−563.659	-	-
**88**	**11.255**	**36,071,721**	**−525.663**	**−62.7**	**−211.4**
**99**	**10.459**	**39,531,328**	**578.098**	**−76.6**	**−226.2**
Total					−211.3

**Table 2:** Model utilization procedure.

**1: while** GPS measurement **do**
**2:** Use true range *r* to obtain transit time *t* by *t* = *r*/*c*,
where *c* is the speed of light in vacuum.
**3:** Use relative received power *p_rel* to obtain actual
received power *p* by *p* = *p_rel* + *p_ref*, where
*p_ref* is the reference power set in the software simulator.
**4:** Obtain differential Doppler *f* by differencing the first satellite with the rest.
**5:** Obtain differential transit time ∆ by differencing the first satellite with the rest.
**6:** Obtain the number of 1ms in ∆, *K* by *K* = floor (absolute value of ∆ × 10^3^).
**7:** Obtain the number of code chips in ∆, *C* by *C* = round( (∆ − *K*) × chip_rate × 10^−3^).
**8: if** *C* is equal to code period (2046) **then**
**9:** reset *C*.
**10:** increment *K*.
**11: end if**
**12:** Use Equations (20) and (23) to obtain *SSC_B_*_1-*I*_.
**13:** *I*_0_ = *p* + *SSC_B_*_1-*I*_.
**14: end while**

**Table 3:** Parameters used in model validation.

Sections	Parameters	Values
Satellite Transmitter (TX)	TX bandwidth *B_T_*	30.69 MHz
	Target signal	BDS B1
Atmospheric Effects, multipath & RF interference	none	Not Applicable
RFFE	Pre-correlation Bandwidth *B_r_*	4 MHz
	Sampling frequency *f_s_*	62 Msps
	Number of quantization levels *N_Q_*	4
	Noise floor	−203.5 dBW/Hz
	Power level at antenna	−160 dBW
Software Receiver	DLL one-sided noise bandwidth *B_L_*	1 Hz
	Coherent Integration Time (CIT) *CT*	20 ms
	Non-coherent Integration Time (NIT) *NT*	1
	Discriminator type	Dot product
	Early-late spacing ∆	0.5 chips

## Appendix A. Autocorrelation Correlation Function Derivations

First, we will derive ACF for the undesired signal. Since the signal is assumed cyclostationary, a two-parameter ACF instead of one-parameter (a single time difference between a stationary signal and its lagged version) ACF is developed here.

### Appendix A.1. Undesired Signal ACF

We start from the definition of two-parameter ACF:

(A1) $$R_{11}\left(s,t\right)≜E\left\{x_{1}\left(s\right)x_{2}\left(t\right)\right\}$$

Substitute Equation (1) into Equation (A1) and we will arrive at:

(A2) $$\begin{array}{c} R_{11}\left(s,t\right)\\ =E\{\sum_{k=-\infty }^{\infty }d_{k}\sum_{u=0}^{19}\alpha_{u}\sum_{v=0}^{2045}c_{v}p_{T_{c}}\left(s-[40,920k+2046u+v]T_{c}\right)\cdot \\ \sum_{n=-\infty }^{\infty }d_{n}\sum_{p=0}^{19}\alpha_{p}\sum_{q=0}^{2045}c_{q}p_{T_{c}}\left(t-[40,920n+2046p+q]T_{c}\right)\}\\ =\sum_{k=-\infty }^{\infty }\sum_{n=-\infty }^{\infty }\sum_{u=0}^{19}\sum_{p=0}^{19}\sum_{v=0}^{2045}\sum_{q=0}^{2045}E\left\{c_{v}c_{q}\right\}\cdot E\left\{d_{k}d_{n}\right\}\cdot \\ \alpha_{u}p_{T_{c}}\left(s-[40,920k+2046u+v]T_{c}\right)\cdot \\ \alpha_{p}p_{T_{c}}\left(t-[40,920n+2046p+q]T_{c}\right)\\ =\sum_{k=-\infty }^{\infty }\sum_{u=0}^{19}\sum_{p=0}^{19}\sum_{v=0}^{2045}\alpha_{u}p_{T_{c}}\left(s-[40,920k+2046u+v]T_{c}\right)\cdot \\ \alpha_{p}p_{T_{c}}\left(t-[40,920k+2046p+v]T_{c}\right)\\ =\sum_{p=0}^{19}p_{T_{c}}\left(t-[40,920k+2046p+v]T_{c}\right) \end{array}$$

where *k* and *v* are selected as:

(A3) $$\begin{array}{l} k=\mathrm{floor}\left(s/T_{b}\right)\\ v=\mathrm{mod}\left(\mathrm{floor}\left(s/T_{c}\right),2046\right) \end{array}$$

such that:

(A4) $$0\leq s-[40,920k+2046u+v]T_{c}<T_{c}$$

We can see from the last line of Equation (A2) that the effects of NH code are cancelled out by the 20 ms integration time. Next, we will derive ACF for the desired signal in a similar way.

### Appendix A.2. Desired Signal ACF

Similar to the process revealed in Equation (A2) through Equation (A4), the receiver local replica (desired signal) ACF could be derived as:

(A5) $$\begin{array}{c} R_{22}\left(s,t\right)\\ =E\{\sum_{k=-\infty }^{\infty }\sum_{u=0}^{2045}c_{k}p_{T_{c}}\left(s-\left[2046k+u\right]T_{c}\right)\cdot \\ \sum_{n=-\infty }^{\infty }\sum_{v=0}^{2045}c_{n}p_{T_{c}}\left(t-\left[2046n+v\right]T_{c}\right)\}\\ =\sum_{k=-\infty }^{\infty }p_{T_{c}}\left(t-\left[2046k+v\right]T_{c}\right) \end{array}$$

where:

(A6) $$v=\mathrm{mod}\left(\mathrm{floor}\left(s/T_{c}\right),2046\right)$$

This ACF for the desired signal will be used in Appendix B where correlator output variance will be derived. This correlator output variance will be transferred to SSC and finally, the analytical form of equivalent white noise, i.e., model for the effects of self-interference will be developed.

## Appendix B. Correlator Output Variance Derivations

We will develop analytical forms of correlator output variance in two situations: (i) data bits are aligned or (ii) data bits are misaligned by an integer number of primary code chips. The third situation where data bits are misaligned by an arbitrary fraction of a primary code chips is dealt with in Section 3.3 “*Data Bits Misaligned by a Fraction of a Code Chip*” of Section 3 “SSC and Equivalent White Noise Level”. For simplicity, we use notation ⌊a⌋ to denote function floor (*a*).

### Appendix B.1. Data Bits Aligned

In this case, the variance at the output of correlator will be:

(A7) $$\begin{array}{l} E\left\{{\left|y_{k}\right|}^{2}\right\}\\ =\int_{kT_{b}}^{(k+1)T_{b}}\int_{kT_{b}}^{(k+1)T_{b}}\sum_{p=-\infty }^{\infty }p_{T_{c}}\left(t-2046kT_{c}-vT_{c}\right)e^{j2\pi fs}e^{-j2\pi ft}dsdt\\ =\int_{kT_{b}}^{(k+1)T_{b}}e^{j2\pi fs}\int_{kT_{b}}^{(k+1)T_{b}}\sum_{p=-\infty }^{\infty }p_{T_{c}}\left(t-2046kT_{c}-vT_{c}\right)e^{-j2\pi ft}dtds \end{array}$$

the inner part of which is a Fourier transform:

(A8) $$F\left[p_{T_{c}}\left(t\right)\right]=T_{c}\sin c\left(fT_{c}\right)e^{-j\pi fT_{c}}$$

and therefore we have:

(A9) $$\begin{array}{l} E\left\{{\left|y_{k}\right|}^{2}\right\}\\ =T_{c}\sin c\left(fT_{c}\right)e^{-j\pi fT_{c}}\sum_{k=0}^{19}e^{-j2\pi fkT}\int_{kT_{b}}^{(k+1)T_{b}}e^{j2\pi f\left(s-vT_{c}\right)}dtds \end{array}$$

where:

(A10) $$\sum_{k=0}^{19}e^{-j2\pi fkT}=\frac{1-e^{-j2\pi fT_{b}}}{1-e^{-j2\pi fT}}$$

where exponential sum formula is applied. Without loss of generality, we focus on the first data bit:

(A11) $$\begin{array}{l} \int_{0}^{T_{b}}e^{j2\pi f\left(s-vT_{c}\right)}ds\\ =\sum_{k=0}^{19}\int_{kT}^{\left(k+1\right)T}e^{j2\pi f\left(s-\left(\left\lfloor s/T_{c}\right\rfloor -2046\left\lfloor s/T\right\rfloor \right)T_{c}\right)}ds\\ =\sum_{k=0}^{19}e^{j2\pi fkT}\sum_{m=0}^{2045}\int_{mT_{c}}^{\left(m+1\right)T_{c}}e^{j2\pi f\left(s-\left\lfloor s/T_{c}\right\rfloor T_{c}\right)}ds\\ =\sum_{k=0}^{19}e^{j2\pi fkT}\sum_{m=0}^{2045}\int_{0}^{T_{c}}e^{j2\pi fs}ds\\ =2046T_{c}\sin c\left(fT_{c}\right)e^{j\pi fT_{c}}\sum_{k=0}^{19}e^{j2\pi fkT} \end{array}$$

which assumes *k* = 0 in Equation (A9), and finally we have:

(A12) $$\begin{array}{l} E\left\{{\left|y_{k}\right|}^{2}\right\}\\ =T_{c}{\left[\sin c\left(fT_{c}\right)\right]}^{2}\cdot \frac{1-e^{-j2\pi fT_{b}}}{1-e^{-j2\pi fT}}\cdot \frac{1-e^{j2\pi fT_{b}}}{1-e^{j2\pi fT}}\\ =T\cdot T_{c}\cdot \frac{\sin^{2}\left(\pi fT_{c}\right)}{{\left(\pi fT_{c}\right)}^{2}}\cdot \frac{\sin^{2}\left(\pi fT_{b}\right)}{\sin^{2}\left(\pi fT\right)} \end{array}$$

which is the variance at the output of correlator. Next, we will derive analytical form of correlator output variance when the data bits are misaligned by an integer number of code chips.

### Appendix B.2. Data Bits Misaligned by an Integer Number of Code Chips

In this case, the variance at the output of correlators will be:

(A13) $$\begin{array}{l} E\left\{{\left|y_{k}\right|}^{2}\right\}\\ =\int_{s=0}^{\Delta }\int_{t=0}^{T_{b}}\sum_{p=0}^{19}p_{T_{c}}\left(t-\Delta -pT+T_{b}-vT_{c}\right)e^{j2\pi f\left(s-t\right)}dtds\\ +\int_{s=\Delta }^{T}\int_{t=0}^{T_{b}}\sum_{p=0}^{19}p_{T_{c}}\left(t-\Delta -pT-vT_{c}\right)e^{j2\pi f\left(s-t\right)}dtds \end{array}$$

whose integration area will be divided into four smaller regions and be dealt with separately to facilitate derivation:

(A14) $$\begin{array}{l} D_{1}=\left\{s:\;0\leq s<\Delta ,\;\mathrm{mod}\left(s,T_{c}\right)<CT_{c}\right\}\\ D_{2}=\left\{s:\;0\leq s<\Delta ,\;\mathrm{mod}\left(s,T_{c}\right)\geq CT_{c}\right\}\\ D_{3}=\left\{s:\;\Delta \leq s<T_{b},\;\mathrm{mod}\left(s,T_{c}\right)<CT_{c}\right\}\\ D_{4}=\left\{s:\;\Delta \leq s<T_{b},\;\mathrm{mod}\left(s,T_{c}\right)\geq CT_{c}\right\} \end{array}$$

This leads to a four-part integration:

(A15) $$\begin{array}{l} E\left\{{\left|y_{k}\right|}^{2}\right\}\\ =\int_{\begin{array}{l} s=0\\ s\in D_{1} \end{array}}^{\Delta }\int_{t=0}^{T_{b}}\sum_{p=0}^{19}p_{T_{c}}\left(t-\Delta -pT+T_{b}-vT_{c}\right)e^{j2\pi f\left(s-t\right)}dtds\\ +\int_{\begin{array}{l} s=0\\ s\in D_{2} \end{array}}^{\Delta }\int_{t=0}^{T_{b}}\sum_{p=0}^{19}p_{T_{c}}\left(t-\Delta -pT+T_{b}-vT_{c}\right)e^{j2\pi f\left(s-t\right)}dtds\\ +\int_{\begin{array}{l} s=\Delta \\ s\in D_{3} \end{array}}^{T}\int_{t=0}^{T_{b}}\sum_{p=0}^{19}p_{T_{c}}\left(t-\Delta -pT-vT_{c}\right)e^{j2\pi f\left(s-t\right)}dtds\\ +\int_{\begin{array}{l} s=\Delta \\ s\in D_{4} \end{array}}^{T}\int_{t=0}^{T_{b}}\sum_{p=0}^{19}p_{T_{c}}\left(t-\Delta -pT-vT_{c}\right)e^{j2\pi f\left(s-t\right)}dtds \end{array}$$

which becomes:

(A16) $$\begin{array}{l} E\left\{{\left|y_{k}\right|}^{2}\right\}\\ =\int_{\begin{array}{l} s=0\\ s\in D_{1} \end{array}}^{\Delta }e^{j2\pi fs}\sum_{p=19-K}^{19}e^{-j2\pi f\left(\Delta +pT-T_{b}+vT_{c}\right)}ds\\ +\int_{\begin{array}{l} s=0\\ s\in D_{2} \end{array}}^{\Delta }e^{j2\pi fs}\sum_{p=20-K}^{19}e^{-j2\pi f\left(\Delta +pT-T_{b}+vT_{c}\right)}ds\\ +\int_{\begin{array}{l} s=\Delta \\ s\in D_{3} \end{array}}^{T}e^{j2\pi fs}\sum_{p=0}^{18-K}e^{-j2\pi f\left(\Delta +pT+vT_{c}\right)}ds\\ +\int_{\begin{array}{l} s=\Delta \\ s\in D_{4} \end{array}}^{T}e^{j2\pi fs}\sum_{p=0}^{19-K}e^{-j2\pi f\left(\Delta +pT+vT_{c}\right)}ds \end{array}$$

The first integration of Equation (A16) is:

(A17) $$\begin{array}{l} \int_{\begin{array}{l} s=0\\ s\in D_{1} \end{array}}^{\Delta }e^{j2\pi fs}\sum_{p=19-K}^{19}e^{-j2\pi f\left(\Delta +pT-T_{b}+vT_{c}\right)}ds\\ =e^{j2\pi fT_{b}}\sum_{p=19-K}^{19}e^{-j2\pi fpT}\int_{\begin{array}{l} s=0\\ s\in D_{1} \end{array}}^{\Delta }e^{j2\pi f\left(s-\Delta -vT_{c}\right)}ds\\ =e^{j2\pi fT_{b}}\sum_{p=19-K}^{19}e^{-j2\pi fpT}\sum_{n=0}^{K}\int_{nT}^{CT_{c}+nT}e^{j2\pi f\left(s-\Delta -vT_{c}\right)}ds\\ =e^{j2\pi fT_{b}}\sum_{p=19-K}^{19}e^{-j2\pi fpT}\sum_{n=0}^{K}e^{j2\pi f\left(n-K-1\right)T}\sum_{m=0}^{C-1}\int_{\left(m-C\right)T_{c}}^{\left(m-C+1\right)T_{c}}e^{j2\pi f\left(\alpha -\left\lfloor \alpha /T_{c}\right\rfloor T_{c}\right)}d\alpha \\ =CT_{c}\frac{\sin\left(\pi fT_{c}\right)}{\left(\pi fT_{c}\right)}e^{j\pi fT_{c}}\frac{\sin^{2}\left(\pi f\left(K+1\right)T\right)}{\sin^{2}\left(\pi fT\right)} \end{array}$$

The second integration of Equation (A16) is:

(A18) $$\begin{array}{l} \int_{\begin{array}{l} s=0\\ s\in D_{2} \end{array}}^{\Delta }e^{j2\pi fs}\sum_{p=20-K}^{19}e^{-j2\pi f\left(\Delta +pT-T_{b}+vT_{c}\right)}ds\\ =e^{j2\pi fT_{b}}\sum_{p=20-K}^{19}e^{-j2\pi fpT}\int_{\begin{array}{l} s=0\\ s\in D_{2} \end{array}}^{\Delta }e^{j2\pi f\left(s-\Delta -vT_{c}\right)}ds\\ =e^{j2\pi fT_{b}}\sum_{p=20-K}^{19}e^{-j2\pi fpT}\sum_{n=0}^{K-1}\int_{CT_{c}+nT}^{\left(n+1\right)T}e^{j2\pi f\left(s-\Delta -vT_{c}\right)}ds\\ =e^{j2\pi fT_{b}}\sum_{p=20-K}^{19}e^{-j2\pi fpT}\sum_{n=0}^{K-1}e^{j2\pi f\left(n-K\right)T}\int_{\left(n-K\right)T}^{\left(n-K+1\right)T-CT_{c}}e^{j2\pi f\left(\alpha -\left\lfloor \alpha /T_{c}\right\rfloor T_{c}\right)}d\alpha \\ =e^{j2\pi fT_{b}}\sum_{p=20-K}^{19}e^{-j2\pi fpT}\sum_{n=0}^{K-1}e^{j2\pi f\left(n-K-1\right)T}\cdot \left(2046-C\right)\int_{0}^{T_{c}}e^{j2\pi f\alpha }d\alpha \\ =\left(2046-C\right)T_{c}\frac{\sin\left(\pi fT_{c}\right)}{\left(\pi fT_{c}\right)}e^{j\pi fT_{c}}\frac{\sin^{2}\left(\pi fKT\right)}{\sin^{2}\left(\pi fT\right)} \end{array}$$

The third integration of Equation (A16) is:

(A19) $$\begin{array}{l} \int_{\begin{array}{l} s=\Delta \\ s\in D_{3} \end{array}}^{T_{b}}e^{j2\pi fs}\sum_{p=0}^{18-K}e^{-j2\pi f\left(\Delta +pT+vT_{c}\right)}ds\\ =\sum_{p=0}^{18-K}e^{-j2\pi fpT}\int_{\begin{array}{l} s=\Delta \\ s\in D_{3} \end{array}}^{T_{b}}e^{j2\pi f\left(s-\Delta -vT_{c}\right)}ds\\ =\sum_{p=0}^{18-K}e^{-j2\pi fpT}\sum_{n=0}^{18-K}e^{j2\pi f\left(n-K\right)T}\int_{\left(n-K\right)T}^{\left(n-K+1\right)T-CT_{c}}e^{j2\pi f\left(\alpha -\left\lfloor \alpha /T_{c}\right\rfloor T_{c}\right)}d\alpha \\ =\sum_{p=0}^{18-K}e^{-j2\pi fpT}\sum_{n=0}^{18-K}e^{j2\pi fnT}\sum_{m=0}^{C-1}\int_{mT_{c}}^{\left(m+1\right)T_{c}}e^{j2\pi f\left(\alpha -mT_{c}\right)}d\alpha \\ =CT_{c}\frac{\sin\left(\pi fT_{c}\right)}{\left(\pi fT_{c}\right)}e^{j\pi fT_{c}}\frac{\sin^{2}\left(\pi f\left(19-K\right)T\right)}{\sin^{2}\left(\pi fT\right)} \end{array}$$

The fourth integration of Equation (A16) is:

(A20) $$\begin{array}{l} \int_{\begin{array}{l} s=\Delta \\ s\in D_{4} \end{array}}^{T_{b}}e^{j2\pi fs}\sum_{p=0}^{19-K}e^{-j2\pi f\left(\Delta +pT+vT_{c}\right)}ds\\ =\left(2046-C\right)T_{c}\frac{\sin\left(\pi fT_{c}\right)}{\left(\pi fT_{c}\right)}e^{j\pi fT_{c}}\frac{\sin^{2}\left(\pi f\left(20-K\right)T\right)}{\sin^{2}\left(\pi fT\right)} \end{array}$$

Finally, we combine Equation (A17) through Equation (A20) and will arrive at:

(A21) $$\begin{array}{l} E\left\{{\left|y_{k}\right|}^{2}\right\}\\ =T_{c}^{2}{\left[\sin c\left(fT_{c}\right)\right]}^{2}\cdot \\ (\left(1023-C\right)\cdot \frac{\sin^{2}\left(\pi fKT\right)+\sin^{2}\left(\pi f(20-K)T\right)}{\sin^{2}\left(\pi fT\right)}\\ +C\cdot \frac{\sin^{2}\left(\pi f\left(K+1\right)T\right)+\sin^{2}\left(\pi f(19-K)T\right)}{\sin^{2}\left(\pi fT\right)}) \end{array}$$

This completes the development of the variance of correlator output when the data bits are misaligned by an integer multiple of primary code chips. Specifically, when *C* = 0, i.e., the differential time delay between undesired and desired signals are integer multiples of a code period, we arrive at:

(A22) $$\begin{array}{l} E\left\{{\left|y_{k}\right|}^{2}\right\}\\ =T\cdot T_{c}{\left[\sin c\left(fT_{c}\right)\right]}^{2}\frac{\sin^{2}\left(\pi fKT\right)+\sin^{2}\left(\pi f(20-K)T\right)}{\sin^{2}\left(\pi fT\right)} \end{array}$$

which is a special case and we put it here for ease of reference.

## Appendix C. Simulation Case For Section “Results”

The receiver is placed at 31°13.3′ N, 121°27.12′ E, Shanghai, China. The TLE retrieved for the simulation results is provided in Table A1.

**Table A1:** TLE for the simulation results in Table 1.

SatelliteNo.	Inclination (degrees)	Right Ascension of the Ascending Node (degrees)	Eccentricity	Argument of Perigee (degrees)	Mean Anomaly (degrees)	Mean Motion (revol. per day)
36287	1.6224	10.9309	0.0003751	185.3235	28.1598	1.00265967
36590	1.5085	29.1093	0.0004961	300.4656	312.7627	1.00277041
36828	54.3192	201.2797	0.0039362	203.8556	127.6677	1.00249186
37210	0.9282	51.9435	0.0005461	156.2428	31.2782	1.00271739
37256	54.0451	320.3427	0.0030637	199.8493	312.3901	1.00256291
37384	56.8805	81.1849	0.0025638	195.5632	159.1983	1.00273265
37763	54.6352	203.5192	0.0035745	204.2305	243.6925	1.00288677
37948	54.1427	319.7862	0.0029394	199.0165	307.8874	1.00268580
38091	1.0914	26.4422	0.0000830	112.2111	164.4866	1.00268964
38250	55.7223	80.5201	0.0023395	212.3740	12.3101	1.86233381
38251	55.6585	80.0001	0.0025721	205.4810	17.6455	1.86234185
38774	54.7564	200.2406	0.0034012	171.3479	188.7571	1.86251530
38775	54.8606	199.7656	0.0019260	217.0437	222.9654	1.86233085
38953	0.1374	20.0179	0.0001222	337.9805	294.8131	1.00272848

For ease of reference, TLE elements used in simulation are extracted and titled.
